# Development and impact of the Iranian hospital performance measurement program

**DOI:** 10.1186/1472-6963-14-448

**Published:** 2014-10-01

**Authors:** Asgar Aghaei Hashjin, Dionne S Kringos, Jila Manoochehri, Aidin Aryankhesal, Niek S Klazinga

**Affiliations:** Department of Public Health, Academic Medical Center (AMC)/University of Amsterdam, Postbox: 22660, 1100 DD Amsterdam, The Netherlands; Department of Health Services Management, School of Health Management and Information Sciences, Iran University of Medical Sciences, Tehran, Iran; Department of Quality Improvement, Tehran Heart Center Hospital, Tehran University of Medical Sciences, Tehran, Iran

**Keywords:** Hospital performance, Performance measurement, Accreditation, External evaluation, Quality assurance, Quality improvement, Iran

## Abstract

**Background:**

Iran developed a national hospital performance measurement program (HPMP) which has been implemented annually throughout its hospitals since 1997. However, little is known yet about its development and the impact of the program on hospital performance.

This study aims to describe the development and process of implementation of the HPMP, and to explore the impact on hospital performance by looking at the trends of performance scores of all different types of Iranian hospitals.

**Methods:**

This was a mixed method study consisting of longitudinal data and qualitative document analysis. Hospital performance data over the period of 2002 to 2008 was analysed.

**Results:**

Iran instituted a comprehensive HPMP and implemented it in all hospitals since 1997. The program followed a phased development to stimulate performance and quality improvement in hospitals. Overall, the program has had a positive impact on the performance of general and specialized hospitals. The performance of general hospitals did not appear to be associated with their size or affiliated university ranking. However, the rate of performance improvement of general teaching and private hospitals was significantly lower than the average improvement rate of all general hospitals. There was no relationship between teaching status of the specialized hospitals and their level of performance. However, the performance of the governmental specialized hospitals showed a substantial decline over time. Moreover, among specialized hospitals, the bigger sized and those affiliated with higher ranked universities, reported better performance.

**Conclusions:**

Overall, the development and implementation of an obligatory HPMP in Iran has improved the level of performance in general and specialized hospitals. However, there is room for further performance improvement especially in the general teaching, private, and governmental specialized hospitals. Reconsidering the ownership type, funding mechanisms and responsibility for the HPMP may have an impact on the absolute level of performance and improvement capacity of hospitals. In addition, the role and composition of survey teams, mechanism of implementation according to the characteristics of hospitals, and updating standards are important factors to promote performance improvement and hospital accreditation requirements.

## Background

Performance and quality improvement (QI) of hospitals has become an intrinsic target of health care systems. Nowadays most health care managers and policy makers are trying to find mechanisms to measure performance and improve quality in health care [1, 2]. Access to health care organizations’ performance and quality information is now considered as an absolute right for communities and patients because they could be better informed for making choices between health care facilities and providers [3]. Performance measurement (PM) is a key concept of QI initiatives; which provides information on the level of achievement of quality improvement targets and facilitates the identification of opportunities for improvement [4–6]. Various studies e.g. [7–12] have reported that PM causes improvements in health care performance; such as quality of care, efficiency and accountability in different sectors of health systems. Hospital PM usually focuses on the level of achievement of specific functional, clinical and administrative targets. It provides information to compare hospital’s commitment with the original targets, standards or expectations which can facilitate the identification of possible opportunities for improvement in different dimensions [4, 13–17].

Considering the important role of PM in the health sector and the increasing interest in development and expansion of PM especially in hospitals, health care systems around the world have invested in measuring and reporting hospital performance data in recent years [18, 19]. As a result, considerable resources are spent on performance and quality measurement and reports which make them as influential tools for policy makers [4, 20, 21]. For example, at international level the World Health Organization (WHO)–among others–reports the results of health systems PM globally [22]. At national level, most recently in 2012, the US government established a bonus and penalty system for hospitals which links the payments to the level of performance and quality of care provided to patients [23].

In response to the worldwide interest and increasing demands for PM, the Iranian Ministry of Health and Medical Education (MOHME) instituted in 1997 an evaluation system for measuring performance of hospitals and improving their quality. The so-called “Hospital Evaluation Program” will be referred to in this paper as “Hospital Performance Measurement Program” (HPMP). The MOHME measures the performance of all hospitals at least once a year by grading them on a six point scale according to their performance scores. The government obliged all hospitals by statutory to undergo the HPMP. The results are linked to the financial mechanisms through a pay for performance (P4P) and performance-based budgeting system [12]. Therefore, the PM has a substantial impact on budget allocation and payments to the hospitals.

Although the Iranian HPMP is one of the first, most comprehensive [20] and unique evaluation programs in the world, little is known about its the development, procedures of implementation phases and impact of the program on the performance of hospitals [24]. Hence in this paper we aim to describe the development and current implementation procedures of the Iranian HPM program, and to explore its impact on the performance of Iranian hospitals by answering the following three questions:

1. How was the HPMP developed and implemented across the Iranian hospitals?
2. What is the trend of Iranian hospitals’ performance results, as assessed through the performance scores, over the period of 2002 to 2008?
3. Is there any association between the type of ownership, teaching status, hospital size or rank of the medical university (to which hospitals are affiliated) of the general (non) teaching and specialized hospitals and the hospitals’ performance scores over the period of 2002 to 2008?

## Methods

We performed a mixed qualitative and quantitative study, consisting of a descriptive analysis of the implementation of the HPM program, and a quantitative comparative analysis of hospital performance data over the period of 2002 to 2008. The descriptive data for this study were collected from accessible official documents of the MOHME issued between 1997 and 2010, medical universities’ websites, and a PhD thesis [12, 25–27]. Professional experiences of the authors as health services researcher and hospital manager were also included. The descriptive data were first verified by experts who were hospital administrators, quality improvement managers and hospital evaluation implementers. The information then was translated from Persian (Farsi) into English by the authors who were experts in health services research and health care management. Finally, the data were checked by five experts who were qualified in both the English language and hospitals affairs. This group controlled the quality of content and translation. The first author was involved in all of the aforementioned steps.

**Figure 1:**
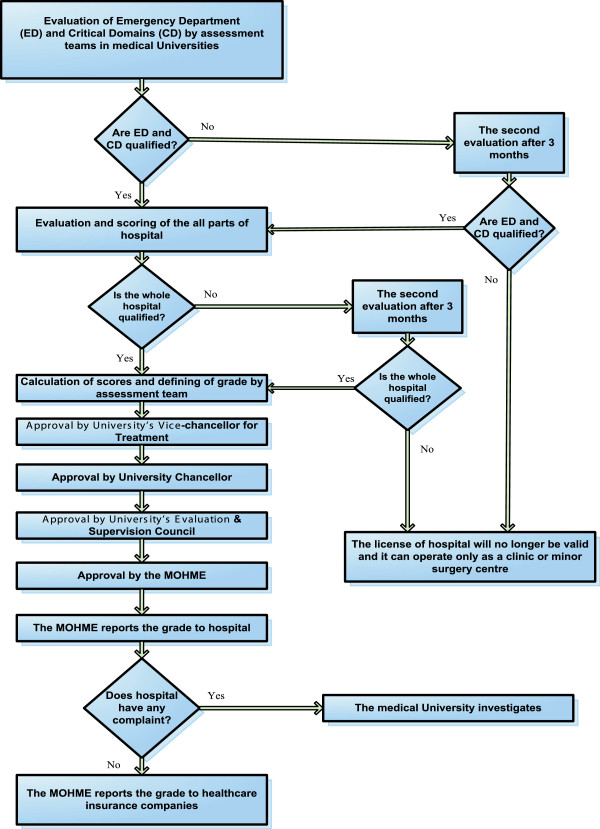
**The flow chart of the process of a typical hospital evaluation & grading in Iranian Healthcare System, adapted from Aryankhesal (2010)** [24]**.** Figure 1 Shows the flow chart of the process of a typical hospital evaluation and grading (scoring) in the Iranian health care system. According to the regulations, hospital emergency departments (ED) and Critical Domains (CD) are evaluated first and only the rest of the hospital would be evaluated if the ED and CD are awarded at least grade 3. If the ED and CD are qualified, the evaluation and scoring of all parts of the hospital will start. If the whole hospital is qualified, calculation of scores and defining the grade will be conducted by an assessment team. The grade as determined by the assessment team should be approved by the university’s Vice-chancellor for Treatment, university Chancellor, university’s Evaluation & Supervision Council and finally by the MOHME.

We analysed the results of specific performance scores of the hospitals to assess the impact of the program using a linear mixed-effects model. The performance scores were measured using the HPM instrument as developed by the Iranian MOHME covering performance, facility and functional domains of quality of care (e.g. safety, patient-centeredness), equipment, manpower, and buildings. Figure 1 shows the flow chart of the process of a typical hospital evaluation and grading (scoring) in the Iranian health care system.

**Figure 2:**
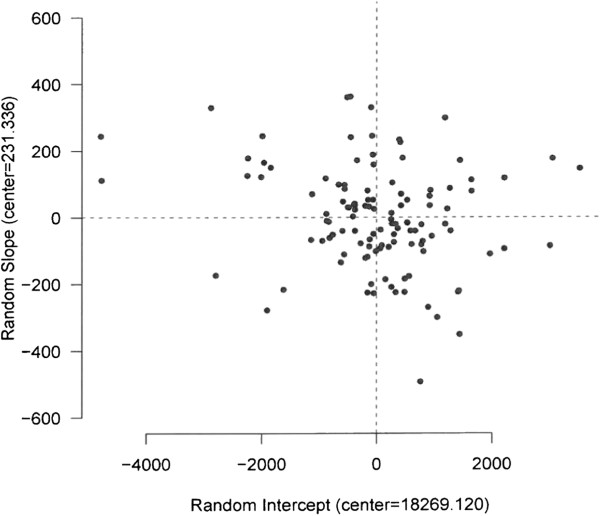
**A linear mixed-effects model for the general teaching hospitals.** The analysis of general teaching hospitals’ performance is shown in Figure 1 (as an example) for the period of 2002 to 2005. The hospitals that are located in the first quadrant (top right), have a higher performance above the average at the baseline. They increased their performance with a higher rate than the average. Quadrant II (top left) represents the hospitals in which their performance was lower than the average performance in the reference year, but the average rate of increase was higher than the overall average. Quadrant III (bottom left) represents the hospitals in which their average performance and the rate of increase both were lower than the overall averages in the reference year. The hospitals in the fourth quadrant (bottom right) showed a higher performance and lower rate of increase than the overall averages.

We made a distinction between multi-specialty and single-specialty hospitals, which we will call general and specialized hospitals respectively in this paper. On average, the performance of 553 general and 143 specialized hospitals were analysed per year over the period of 2002 to 2008 in this study. Different scoring mechanisms in combination with different bonus scores were applied for general teaching and general non-teaching hospitals over the study period. As a result, we studied the performance scores in two different time periods; firstly from 2002 to 2005 and secondly from 2006 to 2008 for both general teaching and general non-teaching hospitals. The most important change for general hospitals took place in 2006 by adding quality indicators to the PM domains, increasing the maximum number of scores which a hospital could achieve with 867. In contrast, a single analysis could be conducted for specialized hospitals for the whole period from 2002 to 2008, due to an absence of critical changes in the scoring/bonus system. The variation (improvement/decline) in performance of specific hospitals from the average performance scores in the reference years (2002 for period 1 and 2006 for period 2) and the average rate of changes in the next years over the study time period were analysed in the model. The model was used to evaluate (graphically) whether hospitals’ performance level (through repeated measurement) changed over the period. In this study, the baseline average performance score in the reference years (intercept) and the mean change (slope) over the period are the fixed effects of the model (which make the center of the effects graph). The random effect is the variability of hospitals from the baseline intercept and slope, which is normally distributed with zero-mean and standard deviation σ. Based on this variability, which allows the hospitals to differ randomly in intercept and slope from the baseline intercept and slope, the hospitals are located in four quadrants in the figure of the effects (see Figure 2).

The quantitative database of hospital performance scores (including grades) over the period of 2002 to 2008 were obtained from the MOHME [28]. We completed the MOHME dataset for our study purposes by including data from two different sources. We included general hospital performance scoring data for the year 2008 from internal reports of the MOHME [29]. The data included the scores of 575 general hospitals affiliated with 37 medical universities across the country in 2008 [30]. The detailed performance scores data were not openly available. We obtained these data from the Department of Evaluation and Supervision of Medical Centers in the MOHME upon the official request provided by the Iran University of Medical Sciences (IUMS). To examine the impact of the national ranking of the medical university on hospitals’ performance, we retrieved the latest Iranian medical universities’ ranking from the openly available report by Mehr (national) News Agency [31].

We followed the RATS guidelines for the qualitative components of the study to ensure all relevant information was included in the manuscript.

The study was approved by the Deputy of Research and Technology of the Iran University of Medical Sciences (Code: 958/1635996). There was no need to obtain informed consent for this study.

## Results

### Hospital performance measurement in Iran

The PM program is a regulatory instrument for quality improvement and quality assurance, and to increase public accountability and informed decision making in hospitals in Iran. The medical universities provide the instructions for PM and standards to all affiliated hospitals in their region, and are responsible for the on-site PM. The program is conducted in a specific period determined by the medical universities in all provinces across the country.

Each medical university has a PM team responsible for conducting evaluations in the affiliated hospitals within a certain province. The teams are composed of at least 10 persons, including at least 2 different medical specialists (internal medicine, general surgery, paediatrics, gynaecology, or anaesthesiology), a radiologist, a clinical technician (preferably a doctor of laboratory sciences or pathologist), an experienced nurse, a medical equipment expert, an inspector for building and construction, an administrative and personnel issues expert, a finance and budgeting expert and a coordinator who is representative of Vice-chancellor for Treatment of the university. Other persons could be included in the team if necessary. Every evaluation team can evaluate at most two hospitals per working week.

According to the regulations, hospital emergency departments (ED) and Critical Domains (CD) are evaluated first and only the rest of the hospital would be evaluated if the ED and CD are awarded at least grade 3. If a hospital does not acquire the necessary scores for its ED and CD, it should improve the situation to the standard level within three months (see Figure 1). In the re-evaluation, if the ED acquires the minimum level of standard, the rest of the hospital will be evaluated. The total hospital grade cannot be better than the awarded grade to the ED which includes 8% of the overall hospital assessment scores [32]. Two different instruments were used for PM of general and specialized hospitals, as described in the next two sections.

#### Performance measurement of general hospitals

The PM instrument for the general teaching and general non-teaching hospitals includes 15 domains with 1027 detailed questions. For each question within a specific domain, a maximum number of scores could be obtained by a hospital. The maximum achievable score for the general teaching and general non-teaching hospitals is 24,667 and 23,667 respectively. The total score for these hospitals can result in one of six grading levels including ‘excellent 1’, ‘1’, ‘2’, ‘3’, ‘4 or sub-standard’, and ‘5 or to be shut down’, based on the hospitals’ degree of compliance with the specified standards of evaluation domains (see Table 1).

**Table 1:** **A) The domains, allocated scores, relevant grades, and B) minimum score requirement for getting specific grade for the general hospitals,** [12, 24–27, 32]

**1A**						
**Domain**		Grade*				
		**Excellent1**	**1**	**2**	**3**	**4 (sub-standard)**
1	***Emergency department (ED)***	1800	1348-1799	899-1347	449-898	Under 448
2	***Sanitation & cleanness***	800	741-799	691-740	640-690	Under 640
3	***Medical records & informatics***	1000	861-999	731-860	600-730	Under 600
4	***Hospital committees***	1000	861-999	731-860	600-730	Under 600
5	***- General quality indicators***	795	596-794	397-595	198-396	Under 198
	***- ED quality indicators***	72	54-71	36-53	18-35	Under 18
6	Consideration of values & Religious regulations	2000	1801-1999	1601-1800	1400-1600	Under 1400
7	Patient satisfaction	1000	861-999	731-860	600-730	Under 600
8	Medical & professional staff	3600	3121-3599	2641-3120	2160-2640	Under 2160
9	Nursing staff	1600	1361-1599	1121-1360	880-1120	Under 880
10	Other staff	1200	1021-1199	851-1020	680-850	Under 680
11	Management	1600	1301-1599	1001-1300	700-1000	Under 700
12	Safety equipment	600	561-599	521-560	480-520	Under 480
13	Non-medical equipment	800	661-799	531-660	400-530	Under 400
14	Medical equipment & medicine	1800	1591-1799	1291-1590	990-1290	Under 990
15	Hospital infrastructure & Installations	2000	1701-1999	1401-1700	1100-1400	Under 1100
**1B**						
Total achievable score**		**(Over) 21667**	**18368-21667**	**15868-18367**	**13219-15867**	**10867-13218**
**Minimum score requirement for getting specific grade**	Teaching hospitals (maximum score = 21667 + 3000 bonus points)	88%	74%	64%	54%	44%
	Non-teaching hospitals (maximum score = 21667 + 2000 bonus points)	92%	78%	67%	56%	46%

* The worst performing hospitals, so-called “to be shut down” with a total score under 10866, for which they are not allowed to undertake any medical activity as a hospital, but as a clinic or minor surgery center.

** There are bonus points for four domains including teaching activities, non-general departments in general hospitals, CCU and ICU and other special facilities; each can add up 500 additional (2000 in total) scores to the non-teaching hospitals’ scores. This bonus can add 3000 scores to teaching hospitals (additional 1000 scores for teaching activities).

In 2002, the MOHME introduced a new domain to the HPMP, with the introduction of nine quality indicators (see Table 2). The new quality indicators domain was introduced with the aim to further improve the quality of care, produce information for decision making, and increase regulation and accountability. The first three indicators were related to the emergency department (ED) with an overall score of 72 (titled ED quality indicators). The remaining six indicators provide the opportunity to assess quality of care in the inpatient services and other sections of hospitals as “general quality indicators”.

**Table 2:** **The quality indicators and relevant scores as subjected to the HPMP from 2002 for general hospitals** [25, 32]

Quality indicator	Maximum score (%)
Waiting time for the first visit of physician in the ED	24 (2.8)
Waiting time for the first nursing services in the ED	24 (2.8)
Patient satisfaction in the ED	24 (2.8)
Pre-operative assessment	50 (5.8)
Pre-operative prophylactic antibiotic therapy	100 (11.5)
Pain management	100 (11.5)
The ratio of caesarean section to natural delivery	140 (16.1)
Safe injections	155 (17.9)
Hospital infections	250 (28.8)
**Total score**	**867 (100)**

In 2006, the MOHME improved the HPMP by re-considering the quality domains; five domains were determined as critical domains (CDs) in the program (as indicated in Table 1A by the first five rows in bold). Obtaining acceptable scores for each of these five domains is a prerequisite for the final evaluation, thus each hospital must acquire a minimum score in any of these domains. In addition, getting an evaluation grade in each grading level requires getting minimum acceptable scores for all of the five domains at that specific level [25].

According to the regulations, hospital EDs are evaluated first. Only when an ED is awarded at least the minimum acceptable score (grade 3), the rest of the hospital is evaluated. According to the regulations, the total hospital grade cannot be better than the awarded grade to the ED which includes 8% of the overall hospital evaluation scores.

#### Performance measurement of specialized hospitals

The performance measurement instrument for the specialized hospitals has 32 domains including 213 questions with related scores for each question which makes the possible maximum: 5688. The total awarded scores for this group of hospitals can result in only four grades including “grade 1”, “2”, “3” and “4 or sub-standard” (Table 3). The process remained unchanged from the official introduction in 1997. The key characteristics of the HPMP in the general and specialized hospitals are shown in Table 4.

**Table 3:** **The domains, allocated scores and relevant grades for performance measurement of the specialized hospitals** [24]

A								
**Domain**		**Score**	**Domain**		**Score**	**Domain**		**Score**
1	Radiology	**925**	12	Kitchen	**113**	23	Central Sterilization Room (CSR)	**50**
2	Management and supervision	**870**	13	Emergency department	**110**	24	Sanitation	**45**
3	Nursing	**720**	14	Using standard forms	**110**	25	Admission and discharge	**43**
4	Engineering and maintenance	**535**	15	Pharmacy	**110**	26	Statistics	**40**
5	Hospital committees	**215**	16	Physiotherapy	**105**	27	Finance	**40**
6	Administration	**215**	17	Medical records	**77**	28	Physician related medical records	**40**
7	Board of physicians	**205**	18	Delivery room	**75**	29	Monthly medical seminars	**30**
8	Ownership	**200**	19	Laundry	**70**	30	Board of directors	**25**
9	Laboratory	**190**	20	Information	**67**	31	Library	**20**
10	Clinics	**180**	21	Board of physicians guideline	**53**	31	Procurement	**20**
11	Operation room	**140**	22	Dialysis unit	**50**	**Total score**		**5688**
**B**								
**Awarded score**				**Grade**		**Minimum score (out of total score) which is necessary for getting specific grade**		
Over 2500				1		44%		
2000-2499				2		35%		
1500-1999				3		26%		
Under 1500				4 (sub-standard)		26%

**Table 4:** **The comparison of obligatory performance measurement (PM) characteristics in general and specialized hospitals**

Type of hospital		Updating of PM program	Number of PM domains	Number of questions in each round of PM	Existence of pre-requisites for PM	Maximum awarded scores in PM	Scales of grading	Responsibility for PM
General (public university, private, SSO and other)	teaching	✓ (Updated in several stages)	14 domains; which increased to 15 domains in 2006	In total 1027 questions in 15 areas	Evaluation of ED was pre-requisite until 2006. From 2006 CD* assessment became obligatory alongside ED	21667 + 3000 bonus points	Six points scale (Excellent1, 1, 2, 3, 4 and 5)	MOHME/ Medical university
	non-teaching	✓ (Updated in several stages)	14 domains;, which increased to 15 domains in 2006	In total 1027 questions in 15 areas	Evaluation of ED was pre-requisite until 2006. From 2006 CD assessment became obligatory alongside ED	21667 + 2000 bonus points	Six points scale (Excellent1, 1, 2, 3, 4 and 5)	MOHME/ Medical university
Specialized (public university, private, SSO and other)		Unchanged (not updated)	32 (remained unchanged)	In total 213 questions in 32 areas	No specific pre-requisites	5688 (no bonus point)	Four points scale (no excellent 1 and 5 scale)	MOHME/ Medical university

*CD = Critical domains.

### The impact of the Iranian hospital performance measurement program

The Iranian HPM program has a direct impact on all hospitals across the country by grading them based on the achieved scores every year. The payments and specific services delivery charges (e.g. patient stay) to the hospitals are defined based on the results of the performance measurement. Hospitals with a better grade (or performance score) are allowed to charge a higher patient stay price. For example hospitals with grade 1 can charge 100% of the patient stay charge per day, while the grade 3 hospitals can charge only 60% of the charges [33].

To explore the impact of the PM program in hospitals, we analysed the trend of performance over the period of 2002 to 2008 by looking at the hospital performance scores. The statistics of hospitals included in the analysis per year are shown in Table 5.

**Table 5:** **The statistics of hospitals included in the study by the type, ownership and year of grading**

**5A) The number of hospitals included by the type, ownership and year of grading in period 1 from 2002 to 2005**												
**Ownership**	**2002**			**2003**			**2004**			**2005**		
	**G.**	**S.**	**Total**	**G.**	**S.**	**Total**	**G.**	**S.**	**Total**	**G.**	**S.**	**Total**
University	296	118	**414**	324	133	**457**	323	135	**458**	343	136	**479**
SSO	47	5	**52**	54	5	**59**	54	4	**58**	57	4	**61**
Private	86	7	**93**	94	10	**104**	99	10	**109**	99	10	**109**
Army	35	5	**40**	38	5	**43**	38	4	**42**	42	5	**47**
Other	44	5	**49**	44	7	**51**	47	9	**56**	45	10	**55**
**Total**	**508**	**140**	**648**	**554**	**160**	**714**	**561**	**162**	**723**	**586**	**166**	**752**
**5B) The number of hospitals included by the type, ownership and year of grading in the period 2 from 2006 to 2008**												
**Ownership**	**2006**			**2007**			**2008**					
	**G.**	**S.**	**Total**	**G.**	**S.**	**Total**	**G.**	**S.**	**Total**			
University	347	136	**483**	339	132	**471**	288	47	**335**			
SSO	59	4	**63**	57	3	**60**	50	0	**50**			
Private	96	9	**105**	93	10	**103**	87	2	**88**			
Army	37	5	**42**	36	4	**40**	36	0	**36**			
Other	45	10	**55**	48	10	**58**	47	0	**47**			
**Total**	**584**	**164**	**749**	**573**	**159**	**732**	**508**	**48**	**556**			

G. = general S. = Specialized.

Among the studied hospitals; 64% were (university) governmental, 8% SSO, 15% private for profit, 6% army, and the remaining 7% belonged to the other organizations.

Table 6 summarises the results of linear mixed-effects model analysis of the general and specialized hospitals' performance scores.

**Table 6:** **The analysis of the trend of hospital performance scores over the period 2002 to 2008**

Hospital		Time period	Average performance score in the first year of time period (intercept)	SD	SE	Average performance score increase per year (slope)	SE	P-value
**General**	Teaching	2002-2005	18269	1367	142.7	231	43.4	0.000
		2006-2008	19557	1235	134.9	180	50.5	5^e^-04
	Non-teaching	2002-2005	16293	1666	79.8	341	20.8	0.000
		2006-2008	18049	1602	75.1	189	25.2	0.000
**Specialized**		2002-2008	2812	648	49.9	46	7.6	0.000

### The relationship between the level of performance and characteristics of general and specialized hospitals

The relationship between the level of performance and characteristics of hospitals including the type of ownership, teaching status, size and national rank of affiliated universities is shown in Table 7. The results show that the performance improvement of general teaching and private hospitals was significantly slower than that of all general hospitals. The performance of the governmental specialized hospitals showed a substantial decline over time. The bigger specialized hospitals (>500 bed) showed significantly faster performance improvement than smaller specialized hospitals. Moreover, the specialized hospitals which were affiliated with the high-ranked universities (such as Tehran, Shiraz and Isfahan) showed a higher performance than specialized hospitals affiliated with lower-ranked universities (such as Ilam, Ghom and Kurdistan).

**Table 7:** The relationship between hospital performance and characteristics of hospitals from 2002 to 2008*

**A) The relationship between hospital performance and ownership of hospitals**									
**Hospital type**	**Time period**	**The average increase/decrease in performance per year by ownership of hospitals (relevant p-values)**						**Total performance increase (p-value)**	
		**Government**	**SSO**	**Private**	**Army**	**Charity**	**Other**		
**General**	2002-2005	24 (0.890)	197 (0.325)	92 (0.621)	97 (0.647)	52 (0.835)	265 (0.384)	317 (0.776)	
	2006-2008	-243 (0.126)	-331 (0.075)	**-345 (0.047)**	-249 (0.214)	81 (0.718)	453 (0.557)	209 (0.118)	
**Specialized**	2002-2008	**-88 (0.038)**	-55 (0.382)	-63 (0.220)	-81 (0.175)	24 (0.729)	126 (0.495)	46 (0.135)	
**B) The relationship between hospital performance and teaching status of hospitals**									
**Hospital type**	**Time period**	**The average performance increase/decrease per year by teaching status of hospitals and relevant p-values**		**Total performance increase (p-value)**					
		Teaching	Non-teaching						
**General**	2002-2005	-118 (0.113)	347 (0.877)	317 (0.113)					
	2006-2008	**-141 (0.044)**	228 (0.929)	**209 (0.044)**					
**Specialized**	2002-2008	-15 (0.307)	53 (0.707)	46 (0.307)					
**C) The relationship between hospital performance and size of hospitals**									
**Hospital type**	**Time period**	**The average performance increase/decrease per year by size (bed number) category of hospitals and relevant p-values**						**Total performance increase (p-value)**	
		**≤100**	**101-200**	**201-300**	**301-400**	**401-500**	**>500**		
**General**	2002-2005	96 (0.703)	-4 (0.987)	133 (0.629)	-118 (0.710)	-142 (0.700)	265 (0.328)	317 (0.608)	
	2006-2008	114 (0.597)	49 (0.824)	124 (0.599)	191 (0.482)	15 (0.962)	103 (0.521)	209 (0.872)	
**Specialized**	2002-2008	-19 (0.699)	-21 (0.663)	-40 (0.495)	-1 (0.991)	-5 (0.945)	**66 (0.042)**	46 (0.986)	
**D) The relationship between hospital performance and national medical universities ranking (which is affiliate the hospital)**									
**Hospital type**	**Time period**	**Total number of hospital (n)**	**The average performance increase/decrease per year by medical university rank and relevant p-values**						**Total performance increase (p-value)**
			**Grade 1**	**n**	**Grade 2**	**n**	**Grade 3**	**n**	
**General**	2002-2005	2214	-78 (0.512)	1127	-22 (0.858)	890	366 (0.912)	193	317 (0.650)
	2006-2008	1666	-78 (0.491)	858	-216 (0.063)	658	331 (0.938)	150	209 (0.053)
**Specialized**	2002-2008	1000	49 (0.053)	577	4 (0.885)	331	16 (0.263)	92	**46 (0.009)**

* Statistically significant relationships are shown in bold.

## Discussion

This study describes the development of the Iranian Hospital Performance Measurement Program and (for the first time) explores its impacts using hospital specific performance scores. The results of the study revealed that the MOHME established an ambitious PM program aimed to improve performance, quality of care, and accountability in hospitals and obliged all hospitals to undergo a scheduled evaluation at least once a year since 1997. Some studies have reported such obligatory HPMP in a limited number of countries [34, 35]. The results show that overall both the general and specialized hospitals improved their level of performance as measured by the program over the study period but the rates of improvement vary in different hospitals. The performance of the general hospitals did not appear to be associated with the ownership, teaching status, size and ranking of the university to which they were affiliated in the period of 2002 to 2005, but the general teaching hospitals performance improved slower than that of general non-teaching hospitals over the period of 2006 to 2008. There was no relationship between teaching status of specialized hospitals and their level of performance. However, the performance of governmental specialized hospitals declined over time. In contrast, the performance of specialized hospitals with more than 500 beds increased significantly faster compared to smaller specialized hospitals. Moreover, the level of performance of specialized hospitals was positively associated with the national ranking of affiliated universities. Although this study provides unique insights into the Iranian hospitals’ performance, it is subject to some limitations concerning the quantitative data used. Underreporting of the hospitals’ performance data to the public by some owners, and limited access to the performance data were the main concerns.

### The Iranian hospital performance measurement program; a unique model for “pay for performance and quality”

The Iranian HPMP is unique in terms of the linkage of the results to the hospital financing (P4P and pay for quality (P4Q); which are both embedded in the program). The PM is on the one hand a regulatory tool for the government to improve the performance and quality of care in hospitals; while on the other hand, it is necessary for the hospitals to get a higher performance score to charge the patient higher and to get a higher share from the annual government budget. The government strictly applies a performance-based budgeting system for the payments to the hospitals based on the results of PM [36]. In addition, the inclusion of quality indicators in the program and establishment of quality evaluation of the hospitals and introduction of “pay and penalty for quality” were the most progressive efforts to push the hospitals being accountable for their performance and the quality of care they provided to patients in Iran [37]. This has been associated with increased efforts among Iranian hospitals to adhere to the audited standards [20, 38]. Recently, the US government disclosed a similar “pay and penalty for quality” method for its Medicare hospitals [23]. Having a higher or lower PM score results in a higher or lower revenue for hospitals. The lower score leads to a lower income and consequently the low rate of functioning could result in a shut down and bankruptcy (especially in the private sector). This can potentially stimulate competition between the hospitals in their ambition to improve their performance and quality of care to get a higher score. As the budget of hospitals and their revenue strongly relate to the PM results, it is recommended that the measurement process runs independently from the owner of hospitals. Performance measurement by an independent body can avoid bias in the results which may originate from the relationship between hospitals and their owners.

### The trend of performance of general and specialized hospitals

#### General hospitals

The performance of the general teaching hospitals improved with an average of 231 points every year from 2002 to 2005. Over the next period (from 2006 to 2008) for the same group of hospitals, the performance scores improved, but the average improvement rate was slower at 180 points per year. The average rate of the performance level of the general non-teaching also increased in from 2002 to 2008. But the rate of improvement was largest over the first period from 2002 to 2005. General teaching hospitals indicated a higher performance in the first time period in 2002, but they improved their performance with a lower rate in the next years until 2006 compared to the general non-teaching hospitals. Over the second time period starting from 2006, although again teaching hospitals showed a higher performance compared to the general non-teaching hospitals, both groups improved their level of performance with almost the same rates in the next years. The average rate of performance improvement of the general non-teaching hospitals was substantially lower in the second time period starting from 2006 compared to the first period. Although both general teaching and general non-teaching hospitals showed an improvement in their performance scores; this can partly be the result of the addition of quality indicators’ scores to the total achievable scores. Another explanation might be the introduction of new medical facilities and not really performance improvement because of the PM program. It seems that getting higher performance scores became more difficult since 2006 onwards for both teaching and non-teaching general hospitals, which may partly be due to the additional critical domains and quality indicators of the PM program and the tightened requirements. There was also a substantial decline in the performance improvement trends of the general teaching and private hospitals in this period.

#### Specialized hospitals

The overall performance of the specialized hospitals improved from 2002 with an average rate of 46 points per year until 2008. However, there is a concern about the governmental specialized hospitals whose performance decreased (on average 88 points per year) compared to the overall improvement rate over the study period. It is difficult to interpret this substantial decreasing performance trend of specialized hospitals. This can be due to several reasons. It can be partly because of the type of ownership and responsibility for the PM among specialized hospitals. The government and medical universities as the owner of a majority (above 80%) of specialized hospitals do not seem to use much force to stimulate the specialized hospitals to increase their level of performance. Using an outdated PM mechanism and minimized requirements for grading of these hospitals indicates a somewhat conservative policy regarding the specialized hospitals by the government. In addition, the quantity of specialized hospitals represents a relatively small segment of the hospital sector in Iran. As a result, it is more difficult for the government to suspend the license of specialised hospitals compared to general hospitals in case of violation from PM requirements.

### The associations between the performance of hospitals and their characteristics

The results show that there was no statistical significant relationship between the level of performance of the general hospitals and the ownership, teaching status, size of hospital and university rank in the period of 2002 to 2005. In contrast, from 2006 to 2008, the average performance improvement of the general teaching hospitals was significantly lower than the overall average rate of improvement. The lower performance improvement of these hospitals may partly relate to the changes in the mechanism of the PM and extra forces by the government on the hospitals to improve their performance and quality of services by tightening the PM standards. Although there was no relationship between the hospitals’ ownership and the level of performance, the performance of the general private hospitals was substantially lower than the overall general hospitals’ performance improvement rate from 2006 to 2008. This might be because of the existence of less commitment with the government to stimulate performance and quality measurement standards in the private hospitals.

The performance of the specialized hospitals was positively related to the size and medical university’s rank, but unrelated to the hospitals’ teaching status. The specialized hospitals with more than 500 beds, showed faster improvement of their performance compared to the smaller specialized hospitals. Moreover, the hospitals which were affiliated with higher ranking universities showed a better annual performance increase rate. This may be because the higher ranked universities were more sensitive to the performance of their specialized hospitals and thus tried to prevent low scores among those hospitals. During the study period, the governmental specialized hospitals’ performance significantly decreased compared to the performance of specialized hospitals which were owned by other organizations. The average performance level of the specialized hospitals in 2002 was around 49% of the maximum possible performance score (5688). Therefore, the majority of hospitals could easily obtain 44% of total achievable performance score which was required to be a grade 1 hospital. On the other hand, a specialized hospital could also achieve 26% of the total score (which is the minimum required score to become a grade 3 hospital) to pass the annual PM and keep its license valid. Therefore, considering the existing requirements for the specialized hospitals to get a grade 1, there seems to be no incentive for the specialized hospitals and even medical universities to improve their performance. Reconsidering the performance grading mechanism and updating the measurement standards may be necessary to encourage the improvement of specialized hospitals’ performance.

### Factors influencing a valid assessment of hospital performance in Iran

Although our study revealed that the overall performance of the Iranian hospitals improved over the study period, it is important to consider the factors that can influence the performance of hospitals when interpreting the hospital performance. The ownership, method of funding, responsibility and validity of the PM are important factors. The PM is conducted by assessment teams which are employed by the medical universities (and the MOHME) which are the owners of hospitals in most cases. Since both universities and hospitals are likely not keen to provide information which might lead to public blame or litigation; the objectivity of the process of PM might potentially be compromised [4]. This potentially also explains why the MOHME has not been very active in publishing the hospitals’ performance data [39]. The dependency of the measurement teams may cause deviation of results in favour of the owners (affiliated medical universities) of hospitals, as suggested by other studies [20, 38]. In many countries, evaluating bodies are usually independent from the ownership of hospital and they are not involved in other processes such as policy making and conducting of PM process [40]. The other important factor is that although the program is unique in terms of the variety of professions involved [41], nonetheless it seems to be necessary to involve different stakeholders including the hospital’s board of directors’ members, hospital managers, patients’ delegates, payers, insurance companies, and quality control officers for standard setting and PM processes. Their involvement in the hospital administration, quality and patient safety issues in the field of hospitals is very useful, making them very familiar with the characteristics of hospital services and performance, and can make professional judgments [42].

Although the scheduled on-site survey is one of the advantages of the Iranian hospital PM program; it could be the case that hospitals prepare themselves briefly in advance and improve their performance just for the period of the conduction of the PM process. This can make the results of the evaluation biased, indicating limited to no sustainable improvements in hospital performance. In addition, as the program is applied both for the teaching and non-teaching hospitals with the same mechanism and standards, applying a PM that is fine-tuned towards the specific characteristics (for example teaching status) of hospitals may increase the accuracy of the measurement results. Finally, the updating of the measurement methods, standards and instruments alongside above mentioned factors may also improve the validity of PM results (as also reported elsewhere [43]). This will also contribute to the development of a mature hospital accreditation model in Iran.

## Conclusion

The Iranian MOHME developed a compulsory national HPMP as early as 1997 and implemented it as a regulatory instrument to improve performance in all hospitals in Iran. Development and implementation of the program for more than one decade shows an extensive effort to establish a framework to improve hospital performance. The existence of such a program has improved the level of performance amongst the Iranian hospitals, but the impact varied in different groups of hospitals. Although in total the performance of both general and specialized hospitals has improved, the improvements in performance scores over time in the general teaching hospitals was substantially lower than that in general non-teaching hospitals. Moreover, the level of performance in the governmental specialized hospitals significantly decreased over the study period. To further promote an effective PM and accreditation model for hospital that meets quality assurance requirements and stimulates the performance improvement efforts in Iranian hospitals it may be helpful to reconsider a number of essential mechanisms that are currently in place. Areas of attention include the roles of different stakeholders in the PM program, the composition of survey teams and their training, ownership and funding of the program. It may also be worthwhile to fine-tune the PM mechanism, procedures of implementation for different hospitals, updating standards and type of scoring systems.

## Authors’ information

**AAH MSc,** 1- Junior Health System Researcher and PhD candidate, Department of Public Health, Academic Medical Center (AMC)/University of Amsterdam, the Netherlands. 2- Researcher in Department of Health Services Management, School of Health Management and Information Sciences, Iran University of Medical Sciences, Tehran, Iran. **DSK PhD,** Postdoctoral Health System Researcher, Department of Public Health, Academic Medical Center, University of Amsterdam, the Netherlands. **JM PhD,** Health System Researcher and quality improvement officer in Tehran Heart Center Hospital, Department of quality improvement (head of department), Tehran University of Medical Sciences, Tehran, Iran. **AA PhD,** Researcher and Assistant Professor of Health Services Management, Department of Health Services Management, School of Health Management and Information Sciences, Iran University of Medical Sciences, Tehran, Iran. **NSK MD PhD,** Professor of Social Medicine, Department of Public Health, Academic Medical Center, University of Amsterdam, the Netherlands.

## Abbreviations

HPMP: Hospital performance measurement program
QI: Quality improvement
WHO: World Health Organization
MOHME: Ministry of health and medical education
P4P: Pay for performance
SSO: Social security organization
ED: Emergency departments
CD: Critical domains
CSR: Central sterilization room
PM: Performance measurement
G: General
S: Specialized
P4Q: Pay for quality.
